# Onco-Hem Connectome—Network-Based Phenotyping of Polypharmacy and Drug–Drug Interactions in Onco-Hematological Inpatients

**DOI:** 10.3390/pharmaceutics18020146

**Published:** 2026-01-23

**Authors:** Sabina-Oana Vasii, Daiana Colibășanu, Florina-Diana Goldiș, Sebastian-Mihai Ardelean, Mihai Udrescu, Dan Iliescu, Daniel-Claudiu Malița, Ioana Ioniță, Lucreția Udrescu

**Affiliations:** 1 Center for Drug Data Analysis, Cheminformatics, and the Internet of Medical Things, Victor Babeş University of Medicine and Pharmacy Timişoara, 300041 Timişoara, Romania; sabina.vasii@umft.ro (S.-O.V.); daiana.handa@umft.ro (D.C.); 2 Doctoral School of Pharmacy, Victor Babeş University of Medicine and Pharmacy Timişoara, 300041 Timişoara, Romania; 3 Department II—Pharmaceutical Chemistry and Biochemistry, Victor Babeş University of Medicine and Pharmacy Timişoara, 300041 Timişoara, Romania; 4 Department of Computer and Information Technology, Politehnica University Timişoara, 300223 Timişoara, Romania; sebastian.ardelean@cs.upt.ro (S.-M.A.); mihai.udrescu@cs.upt.ro (M.U.); 5 Department of Surgery I—Clinic of Surgical Semiotics & Thoracic Surgery, Center for Hepato-Biliary and Pancreatic Surgery, Victor Babeș University of Medicine and Pharmacy Timişoara, 300041 Timişoara, Romania; dan.iliescu@umft.ro; 6 Department XV—Orthopedics-Traumatology, Urology, Radiology and Medical Imaging, Victor Babeș University of Medicine and Pharmacy Timişoara, 300041 Timişoara, Romania; malita.daniel@umft.ro; 7 Multidisciplinary Research Center for Malignant Hemopathies, Victor Babeş University of Medicine and Pharmacy Timişoara, 300041 Timişoara, Romania; ionita.ioana@umft.ro; 8 First Department of Internal Medicine, Victor Babeş University of Medicine and Pharmacy, 300041 Timișoara, Romania; 9 Department of Hematology, Emergency Municipal Hospital, 300254 Timișoara, Romania; 10 Department I—Clinical Pharmacy and Drug Analysis, Victor Babeş University of Medicine and Pharmacy Timişoara, 300041 Timişoara, Romania

**Keywords:** patient similarity network, drug–drug interactions, polypharmacy, hemato-oncology, chemotherapy regimens, network phenotyping

## Abstract

We introduce the Onco-Hem Connectome (OHC), a patient similarity network (PSN) designed to organize real-world hemato-oncology inpatients by exploratory phenotypes with potential clinical utility. **Background:** Polypharmacy and drug–drug interactions (DDIs) are pervasive in hemato-oncology and vary with comorbidity and treatment intensity. **Methods:** We retrospectively analyzed a 2023 single-center cohort of 298 patients (1158 hospital episodes). Standardized feature vectors combined demographics, comorbidity (Charlson, Elixhauser), comorbidity polypharmacy score (CPS), aggregate DDI severity score (ADSS), diagnoses, and drug exposures. Cosine similarity defined edges (threshold ≥ 0.6) to build an undirected PSN; communities were detected with modularity-based clustering and profiled by drugs, diagnosis codes, and canonical chemotherapy regimens. **Results:** The OHC comprised 295 nodes and 4179 edges (density 0.096, modularity *Q* = 0.433), yielding five communities. Communities differed in comorbidity burden (Kruskal–Wallis $\varepsilon^{2}$: Charlson 0.428, Elixhauser 0.650, age 0.125, all FDR-adjusted *p* < 0.001) but not in utilization (LOS, episodes) after FDR ($\varepsilon^{2}$ ≈ 0.006–0.010). Drug enrichment (e.g., enoxaparin Δ = +0.13 in Community 2; vinblastine Δ = +0.09 in Community 3) and principal diagnoses (e.g., C90.0 23%, C91.1 15%, C83.3 15% in Community 1) supported distinct clinical phenotypes. Robustness analyses showed block-equalized features preserved communities (ARI 0.946; NMI 0.941). Community drug signatures and regimen signals aligned with diagnosis patterns, reflecting the integration of resource-use variables in the feature design. **Conclusions:** The Onco-Hem Connectome yields interpretable, phenotype-level insights that can inform supportive care bundles, DDI-aware prescribing, and stewardship, and it provides a foundation for phenotype-specific risk models (e.g., prolonged stay, infection, high-DDI episodes) in hemato-oncology.

## 1. Introduction

Clinical pharmacists are healthcare professionals with a critical role in multidisciplinary cancer care teams by cooperating closely with physicians to optimize drug therapies and prevent drug–drug interactions (DDIs) [1,2]. In hemato-oncology, patients often have comorbidities and treatment-induced toxicity; therefore, pharmacists support chemotherapy preparation, drug reconciliation, electronic knowledge bases, and targeted patient counseling to improve efficacy and safety [1,3,4].

DDIs are particularly significant when drugs have a narrow therapeutic window; modest shifts in exposure may produce either therapeutic failure or toxicity [5]. Although many interactions can be mitigated through monitoring of clinical status and laboratory parameters—and by accounting for patient factors such as age, sex, renal and hepatic function—effective systems also require intuitive, pharmacist-informed electronic alerts embedded in clinical workflow [6,7].

Polypharmacy is common in elderly cancer patients, driven by comorbidities, symptom control, and supportive care for treatment side effects; this markedly increases DDI risk [8,9]. Use of complementary and alternative medicines (CAM)—often without disclosure—further complicates safety; agents such as St. John’s Wort or garlic can induce or inhibit cytochrome P450 pathways, altering exposure to anticancer and supportive therapies [6,10,11]. Because we did not systematically record exposure to CAM in our dataset, it was only included if explicitly documented in inpatient medication lists. In addition to patient factors such as mucositis, malnutrition, edema, and organ dysfunction that affect absorption, distribution, metabolism, and excretion [12], real-world inpatient hemato-oncology practice incorporates multiple supportive medications; this increases both pharmacokinetic and pharmacodynamic interactions [10,13].

In clinical practice, pharmacokinetic DDIs frequently arise between supportive drugs and antineoplastic agents (e.g., warfarin with gemcitabine; QT-prolonging combinations such as doxorubicin with ondansetron), as do pharmacodynamic interactions (e.g., enhanced myelosuppression with cyclophosphamide plus allopurinol) [9]. Some drug combinations are intentionally used despite associated risks (e.g., cisplatin with furosemide to protect renal function); this clinical approach emphasizes that the significance of a DDI depends on the indication, timing of administration, and monitoring practices [9]. Supportive drugs (e.g., antiemetics, antifungals, corticosteroids, and antibiotics) contribute significantly to clinically relevant DDIs and pharmacist interventions, while cardiovascular and diuretic agents are often involved in preventable adverse events [14,15,16]. Given the risk of QTc prolongation (e.g., granisetron with metoclopramide or fluoroquinolone combinations), ECG surveillance is often warranted [3].

In this context, patient-level approaches that integrate diagnoses, comorbidities, medication exposures, and interaction burden can provide a practical, clinically interpretable view of risk. Network-based methods, specifically patient similarity networks, represent heterogeneous data as connections between clinically similar patients, supporting phenotype discovery and targeted stewardship.

In this paper, we introduce the Onco-Hem Connectome (OHC), a PSN built from a 2023 hemato-oncology inpatient cohort. The network integrates demographics, validated comorbidity indices (Charlson and Elixhauser), polypharmacy burden (comorbidity-polypharmacy score—CPS), aggregate DDI severity score (ADSS), diagnoses, and detailed medication patterns. Our objectives are to (1) build a PSN to identify clinically coherent communities (phenotypes), (2) characterize community-specific comorbidity, polypharmacy, DDI patterns, and canonical chemotherapy/supportive care regimens, and (3) propose phenotype-informed decision support hypotheses for prospective evaluation.

## 2. Materials and Methods

### 2.1. Study Design, Ethical Approval, and Data Source

We conducted a retrospective observational study using an electronic hospitalization dataset of successive inpatient episodes for onco-hematological patients admitted between January and December 2023 to Timișoara Municipal Emergency Clinical Hospital. The study was approved by the Scientific Research Ethics Committee of the “Victor Babeș” University of Medicine and Pharmacy, Timișoara (approval no. 56/05.12.2022). We included patients aged ≥18 years who provided informed consent and had at least two drugs recorded.

We used the hospitalization episode as the initial unit of analysis, where a row is for a hospitalization period and a unique patient identification number. For each episode, the dataset includes demographic data (age, sex), admission and discharge dates, ICD-10-coded diagnoses [17], and in-hospital drug treatments. Our dataset comprises 1158 hospital episodes corresponding to 298 unique patients.

### 2.2. Data Preprocessing and Aggregation

We compiled all parameters recorded during each patient visit at the individual patient level.

We used the admission and discharge (inclusive) dates to calculate the length of stay in calendar days; then, we computed for each patient the total number of hospitalizations, total length of stay, and mean length of stay. From the age recorded at the episode level, we derived the minimum, maximum, and mean age at the patient level. Diagnoses were processed by combining the principal diagnosis and the additional diagnoses into a unique list of ICD-10 codes per episode, ensuring there were no duplicates. We used these lists to derive comorbidity scores.

Data management and preprocessing were performed using Microsoft Excel. Subsequent structured data processing, similarity modeling, and statistical analyses were conducted in Python 3.12.7. Specifically, we used Pandas 2.2.2 and NumPy 1.26.4 for data manipulation and matrix operations. High-dimensional feature scaling and the calculation of the cosine similarity matrix were implemented using SciKit-Learn 1.5.1 and SciPy 1.13.1. Inferential statistical testing and clinical characterization of the phenotypes were performed using the statsmodels 0.14.2 library. Network visualization and modularity-based community detection were carried out in Wolfram Mathematica 13.1.

### 2.3. Comorbidity and Polypharmacy Scores

At episode level, we calculated the Comorbidity–Polypharmacy Score (CPS) and a categorical CPS level variable. CPS is the sum of the number of comorbidities and the number of drugs. The CPS level classifies CPS into four risk categories: mild for 0–7 points, moderate for 8–14 points, severe for 15–21 points, and morbid for 22 points or more [18]. At the patient level, CPS was summarized as minimum, maximum, and mean, and CPS level was defined as the most frequent category across episodes.

Using ICD-10 codes, we implemented a simplified mapping to Charlson and Elixhauser comorbidity categories. For the Charlson index, we identified the major 17 comorbidity groups (e.g., heart failure, COPD, diabetes, chronic kidney disease, chronic liver disease, solid malignancy, metastases), then computed the Charlson Comorbidity Index (CCI) score per episode, which was then aggregated at the patient level (mean, minimum, maximum) [19,20]. For Elixhauser, we defined binary flags at the episode level for relevant conditions (e.g., heart failure, COPD, hypertension, obesity, depression, renal disease, liver disease, malignancy, metastases) [21]. Thus, we obtained a simple Elixhauser sum score as the sum of present flags per episode. At the patient level, we derived both an average Elixhauser sum and “ever” flags (1 if the condition appeared in at least one hospitalization), resulting in a binary comorbidity profile for each patient.

### 2.4. Drug Processing, Drug–Drug Interactions, and DDI Severity Score

We used the DrugBank API version 5.1.11 (2024) to assess the severity of drug–drug interaction (DDI) [22]. Since our inpatient dataset provided drug lists at the episode level without intra-episode administration times, we defined DDIs based on the co-presence of two drugs during the same hospitalization episode. For each episode, we recorded the number of minor, moderate, and major DDI. We then calculated the aggregate DDI severity score (ADSS) using the formula: (number of major DDIs × 3) + (number of moderate DDIs × 2) + (number of minor DDIs). Finally, we aggregated this score at the patient level.

We harmonized spelling, case, drug synonyms, and enforced fixed combinations (e.g., sulfamethoxazole and trimethoprim as sulfamethoxazole+trimethoprim) before computing global and community drug prevalences.

For use in the similarity network, we built a binary patient-by-drug matrix in which a value of 1 indicated that the patient had received a given drug at least once during any hospitalization.

### 2.5. Building the Patient Similarity Network (PSN)—Onco-Hem Connectome

At the patient level, we assembled a feature vector that integrates:

- aggregated continuous variables (mean age, mean CPS, mean Charlson and Elixhauser scores, mean ADSS, mean and total length of stay, number of hospitalizations),
- sex (encoded as a binary variable, woman/man),
- Elixhauser comorbidity flags (0/1, ever present),
- medication exposure (the binary patient-by-drug matrix).

Continuous variables were standardized (mean 0, standard deviation 1) while binary variables were kept as 0/1. These concatenated feature vectors were used to quantify similarity between patients.

Patient–patient similarity was defined as the cosine similarity between feature vectors. We utilized cosine similarity over alternative metrics (e.g., Jaccard or Gower) as it effectively handles high-dimensional, mixed feature spaces by focusing on the angular alignment of patient profiles, which is more robust to variations in absolute feature frequency [23,24]. We computed a full similarity matrix in which each entry reflects how similar two patients are in terms of demographics, comorbidity and interaction scores, Elixhauser comorbidities, and medication patterns. From this matrix, we derived a Patient Similarity Network (PSN), an undirected graph in which nodes represent patients and edges connect pairs of patients with a cosine similarity of at least 0.6. This means that an edge is created between two patient nodes when their clinical, pharmacological, and comorbidity profiles—represented as standardized feature vectors—have a sufficiently high cosine similarity (≥0.6); this threshold typically indicates that the patients share similar ages and scores, as well as a significant overlap in their drugs and comorbidities. This choice aligns with established heuristics in patient-centric data analysis where 0.6 is used to differentiate significant clinical associations from background noise [25]. We visualized the PSN from its edge list (patient *i*, patient *j*, similarity) in Wolfram Mathematica 13.1 using an energy-based layout. Community structure was then identified by modularity maximization with FindGraphCommunities (Method -> “Modularity”, greedy modularity maximization in the sense of Clauset–Newman–Moore), and the resulting partition’s modularity (Q) was computed with CommunityModularity.

No predefined clinical inclusion or exclusion criteria were applied to define the patient communities, as group membership emerged from an unsupervised, data-driven patient similarity network and modularity-based community detection.

In exploratory comparisons between patient communities, we applied the Kruskal–Wallis test to continuous variables—age, CPS (mean), Charlson (mean), Elixhauser sum (mean), length of stay (mean), number of episodes, and ADSS (mean); effect sizes are reported as $\varepsilon^{2}$. Categorical variables—sex and CPS level—were compared using the chi-square test of independence with Cramér’s *V* as effect size. We controlled the false discovery rate using the Benjamini–Hochberg procedure, defining families as (i) all continuous omnibus tests and (ii) all categorical omnibus tests.

We characterized the PSN using several network parameters, including node and edge counts, density, edge weight, degree, and strength distributions, and Newman–Girvan modularity [26]. We compared within- and between-community cosine similarities using Mann–Whitney U with Cliff’s δ as effect size. Additionally, we quantified layout similarity concordance via Spearman correlation between similarity and negative 2D layout distance.

To quantify feature-block contributions and to demonstrate that the PSN communities are not simply driven by high-dimensional drug overlap, we also performed attribution and ablation analyses. We partitioned the feature space into a drug block (binary drug exposures) and a non-drug block (age, sex, CPS, Charlson, Elixhauser sum, and ADSS). First, we computed block-specific cosine similarity matrices and related each to the full similarity via a Mantel-type correlation (Spearman on upper-triangle entries) and an edge-level standardized regression of the full similarity on the two block similarities to obtain standardized coefficients (block weights). Second, we rebuilt the PSN under three variants: medications-only, no-medications, and block-equalized weighting (each block scaled so its average L2 contribution is equal), re-clustered each variant with the same procedure as baseline, and compared partitions using Adjusted Rand Index (ARI; chance-adjusted pairwise agreement, range −1 to 1) and Normalized Mutual Information (NMI; information-theoretic overlap, range 0 to 1). For comparability, we also reported modularity *Q* on unweighted graphs obtained by thresholding similarity at 0.60. Stability of the block-equalized variant and only partial recovery under the two ablations suggest that the communities reflect broader clinical structure and are not determined exclusively by the dimensionality of the drugs.

### 2.6. Chemotherapy Regimens

We reconstructed standard chemotherapy regimens using episode-level drug lists; the core components of these regimens are: R-CHOP (rituximab, cyclophosphamide, doxorubicin, vincristine, plus a corticosteroid recorded as prednisone or prednisolone), ABVD (doxorubicin, bleomycin, vinblastine, dacarbazine), and VAD (vincristine, doxorubicin, dexamethasone). We consider a patient to be exposed if their medical record includes all core components during at least one hospitalization in 2023. To accommodate documentation gaps (e.g., steroid recorded as prednisolone, or a single missing line in pharmacy records), we used a pre-specified relaxed rule allowing one missing component when the remaining components were present in the same episode. We summarized prevalence at the patient level and reported it by OHC communities.

## 3. Results

### 3.1. Cohort Description

The dataset includes 298 unique hemato-oncology patients, 154 men and 144 women, with a total of 1158 hospital episodes. Table 1 summarizes patient age, comorbidity, and polypharmacy scores (CPS, Charlson, Elixhauser), the drug–drug interaction burden (ADSS), and the number of episodes per patient, reported as count, mean, standard deviation, minimum, maximum, and interquartile range (IQR).

Our cohort has a predominantly older age profile, with a median age of 65 years (IQR 53–72.96, range 20–91.33), indicating that most patients are late middle-aged to elderly. The overall levels of comorbidity and polypharmacy are high, as illustrated by a median CPS mean of 23 (IQR 19–28.9, range 8–56), a median Charlson score mean of 1.0 (IQR 0–2.0, range 0–5.6), and a median Elixhauser simple sum mean of 4 comorbidities (IQR 2.35–8.0, range 0–21.33). These findings indicate that most patients have multiple chronic conditions and receive many concurrent drugs, with a substantial subset experiencing very high levels of multimorbidity. The DDI burden is also considerable, with a median ADSS mean of 38.0 (IQR 20.08–59.78, range 0–268), indicating that some patients experience extremely high levels of cumulative DDI severity. The healthcare utilization data reveal that the patients had a median of 4 hospitalizations each (IQR 2–6, range 1–12); this outcome confirms the high complexity of our inpatient cohort, which is characterized by older age, substantial comorbidity, intensive pharmacotherapy, and frequent re-hospitalizations.

### 3.2. Onco-Hem Connectome

**Figure 1:**
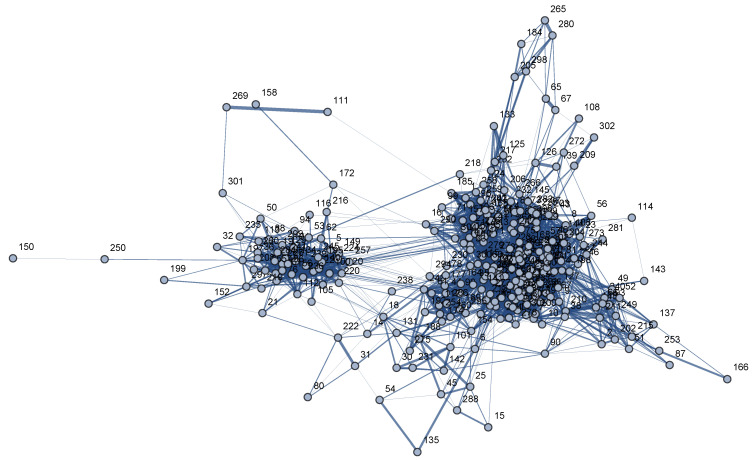
Weighted Onco-Hem Connectome: nodes are represented by patients and are labeled with the patient’s id; an edge between 2 nodes represents the similarity relationship between the 2 corresponding patients and is weighted with the cosine similarity.

In the resulting graph—Onco-Hem Connectome, see Figure 1—295 of the 298 node-patients had at least one edge above the similarity threshold and were integrated into the network. Figure 1 reports the empirical distribution of cosine similarities for the retained edges (≥0.6), which quantifies the similarity scale of observed links and highlights the degree of sparsification caused by thresholding. Three patients had similarity values < 0.6 with all others; as a result, they had no edges in the PSN and were excluded from network detection.

**Figure 2:**
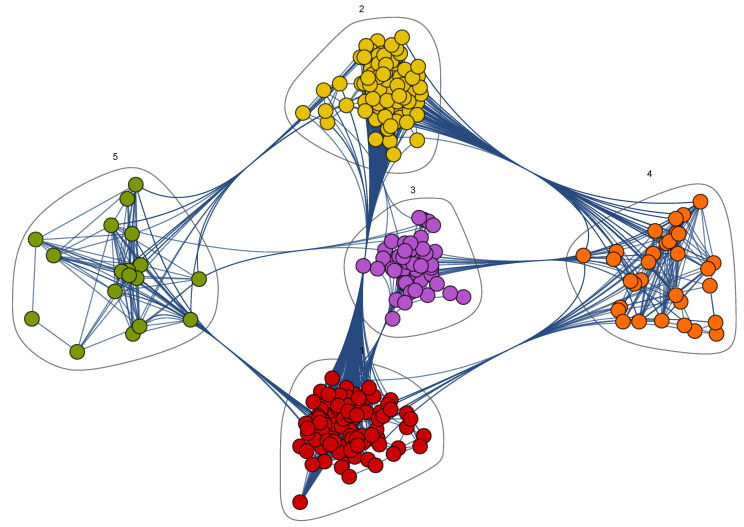
Clustered Onco-Hem Connectome: nodes are grouped into 5 communities or patient phenotypes. Each community has a distinct modularity class (color).

Upon running the Mathematica clustering algorithm, the Onco-Hem Connectome network revealed five distinct communities, each community representing a group of patients with similar clinical and pharmacological profiles, as illustrated in Figure 2.

Table 2 displays the demographic and score profile for each community (number of patients, mean age, sex distribution, mean CPS, Charlson, Elixhauser, ADSS, mean length of stay, mean number of hospitalizations, and CPS level distribution).

Table 3 summarizes the omnibus comparisons, showing significant heterogeneity for age, CPS (mean), Charlson (mean), and Elixhauser sum (mean), while LOS, number of episodes, and ADSS (mean) were not significant after FDR. CPS level distributions also differed, whereas sex did not.

We also compiled a drug profile by determining each drug’s prevalence within its respective community. To highlight drugs that differentiate communities rather than ubiquitous supportive treatments, we excluded drugs with global prevalence > 80% from comparative analyzes. For the remaining drugs, we computed the difference between community-specific and global (cohort-wide) prevalence. Table 4 reports, for each community, the ten drugs with the largest positive differences (and community prevalence ≥ 10%), together with their global prevalence, community-specific prevalence, and the corresponding prevalence difference; these drugs can be interpreted as being over-represented in that community.

Table 5 summarizes, for each Onco-Hem community, the three most frequent principal diagnoses, which are fully consistent with the clinical context of the dataset (hemato-oncology patients hospitalized in a hematology ward). In our cohort, the principal diagnoses that dominate are multiple myeloma (C90.0), diffuse large B-cell lymphoma (C83.3), and acute myeloblastic leukemia (C92.0); all of these appear systematically among the top principal diagnoses in every community.

Table 5 provides descriptive context and summarizes the overall case-mix across the five communities, supporting comparability of the cohort structure. In contrast, Table 6 focuses on the community-specific patterns that drive phenotype differentiation.

To better capture the complexity of the clinical profile in terms of comorbidities (i.e., diseases associated with the underlying hemato-oncologic condition), we quantified the prevalence of additional ICD-10 diagnoses within each OHC community. Table 6 presents, for each community, the three most frequent additional diagnoses (non-principal) ICD-10 diagnoses, together with their patient-level proportions, illustrating how specific comorbidity patterns cluster across the network. Across all communities, immunodeficiency (D84.9) was almost universal and was consistently accompanied by COVID-19–related codes (U07.2), follow-up/screening encounters (Z11.5), essential hypertension (I10), very high rates of anemia in neoplastic disease (D63.0), mitral insufficiency (I34.0), and opportunistic mycoses (B48.7). This pattern confirms that all communities are composed of highly immunocompromised, multimorbid hemato-oncology inpatients with substantial cardiovascular and infectious comorbidity.

The OHC comprised 295 patients connected by 4179 edges at cosine ≥ 0.6 (density 0.096). Edge weights showed a two-component log-normal mixture; degree and strength followed negative-binomial and Weibull forms, respectively. A modularity value *Q* > 0.3 usually indicates significant community structure [26,27]; indeed, a *Q* = 0.433 modularity for the graph partitioning (i.e., node communities) we present in the paper indicates robust community structure. Importantly, within-community similarities were markedly higher than between-community similarities (mean 0.221 vs. −0.0048; median 0.275 vs. −0.024; Mann–Whitney U *p* < $10^{-300}$; Cliff’s δ ≈ 0.28), supporting internal cohesion. To prevent misinterpretation of the visualization, we quantified the association between 2D layout distance and cosine similarity: Spearman ρ = 0.818, *p* < $10^{-300}$ (using −distance so that larger values indicate closer nodes), confirming a strong qualitative correspondence while noting that the layout remains illustrative only.

We quantified block contributions and performed ablations to test whether high-dimensional drug exposure dominates the OHC. First, block-specific cosine similarities correlated strongly with the full similarity for both drugs (ρ = 0.656) and non-drug features (ρ = 0.807; both *p* < $10^{-300}$).

Edge-level standardized regression of full similarity on block similarities yielded $\beta_{\mathrm{non}\text{-}\mathrm{drug}}=0.761$ and $\beta_{\mathrm{drug}}=0.510$, indicating that non-drug features carry the larger share of explanatory weight. Second, we rebuilt and re-clustered the PSN under drugs-only, no-drugs, and block-equalized weighting, ensuring that each block is scaled to have an equal average L2 norm. Compared with the baseline communities, block-equalized labels were highly stable (ARI = 0.946, NMI = 0.941), whereas drug-only and no-drugs recovered only part of the structure (ARI = 0.189, NMI = 0.224, and *Q* = 0.177 compared with ARI = 0.401, NMI = 0.470, and *Q* = 0.068, respectively). Modularity on the thresholded graphs remained of similar magnitude across baseline and block-equalized variants (*Q* = 0.106 vs. 0.102), supporting robustness. These results show that drugs contribute a meaningful signal but do not drive the communities; the phenotypes reflect broader clinical structure dominated by non-drug features.

### 3.3. Chemotherapy Regimen Prevalence

Table 7 presents the standard chemotherapy prevalence across the OHC communities. The ABVD regimen was prevalent in Community 3 (i.e., 13.1%) and was low or absent elsewhere. R-CHOP had moderate overall usage: 9.1% in Community 1, 6.5% in Community 4, and ≤3.5% in others. The VAD regimen showed the highest prevalence of 29% in Community 4; it was also substantially used in Communities 1–3 (17.4–21.3%) and had the lowest presence in Community 5 (5.6%).

### 3.4. Onco-Hem Connectome Phenotypes

The five communities identified displayed distinct clinical and pharmacotherapeutic profiles (Figure 2, Table 2). By integrating patient demographics, comorbidity and polypharmacy metrics (CPS, Charlson, Elixhauser), drug–drug interaction burden (ADSS), medication enrichment patterns (Table 4), principal and additional ICD-10 diagnoses (Table 5 and Table 6), and exposure to canonical chemotherapy regimens (Table 7), we derived the following clinically coherent phenotypes:

- **Community 1—Mixed myeloma/lymphoma phenotype with predominant supportive care pattern.** Community 1 included 99 patients (53% men), mean age 63.38 years, with mean LOS 5.47 days and 3.82 episodes per patient. Comorbidity and polypharmacy were moderate–high (CPS 23.88; Charlson 1.1; Elixhauser 6.79), and the DDI burden substantial (ADSS 41.96); nearly half were CPS Level 4. Principal diagnoses were dominated by multiple myeloma (C90.0, 23%), chronic lymphocytic leukemia–CLL (C91.1, 15%), and diffuse large B-cell lymphoma–DLBCL (C83.3, 15%), while additional diagnoses highlighted a quasi-universal immunocompromised background (D84.9, 92%) alongside hypertension (I10, 88%) and COVID-19-related codes (U07.2, 76%). The top over-represented drugs reflected supportive and regimen-adjacent care rather than a single signature protocol—acetaminophen and filgrastim (Δ = +0.04 each), desloratadine (+0.02), tramadol (+0.01) and arginine/zoledronic acid/spironolactone (+0.01 each), alongside selective antineoplastic (obinutuzumab, doxorubicin) and ciprofloxacin (+0.02). Overall, Community 1 represents a heterogeneous, mid-complexity hemato-oncology cluster (myeloma/lymphoma-centric) managed with broad supportive care and intermittent cytotoxic/immunotherapy, consistent with its intermediate comorbidity and DDI profiles.
- **Community 2—Older, highly multimorbid thrombo–infectious phenotype**. Community 2 (*n* = 86) is the oldest subgroup (mean age 68.26, 56% men) and the most multimorbid (CPS 29.06; Charlson 2.11; Elixhauser 9.77), with high DDI burden (ADSS 47.87) and greater utilization (LOS 6.94 days). Predominant principal diagnoses are multiple myeloma (C90.0, 22%), CLL (C91.1, 13%) and other specified types of non-Hodgkin lymphoma (C85.7, 13%). Additional diagnoses reveal a dense cardio-infectious profile: near-universal immunodeficiency (D84.9, 92%), very high hypertension (I10, 85%), valvular disease (I34.0, 76%), heart failure (I50.9, 59%), postprocedural cardiac complications (I97.9, 57%), plus COVID-related and screening codes (U07.2, 73%; Z11.5, 72%). Drug enrichment aligns with this burden: enoxaparin (Δ = +0.13), acyclovir (+0.12), diuretics such as furosemide (+0.11) and spironolactone (+0.05), antibacterials co-trimoxazole (sulfamethoxazole+trimethoprim, +0.07) and meropenem (+0.06), alongside metamizole, lidocaine, alprazolam, and rituximab—a pattern consistent with thrombo-prophylaxis, anti-infective prophylaxis/therapy, and volume/arrhythmia and analgesia management accompanying hematologic treatment.
- **Community 3—Younger chemo-intensive leukemia and lymphoma phenotype**. Community 3 (n = 61, 51% men) is the youngest group and displays the lowest non-malignant comorbidity burden (Charlson 0.07, Elixhauser 0.28) but still considerable polypharmacy and DDI exposure (CPS 21.54, ADSS 46.08). Principal diagnoses are dominated by acute myeloid leukemia (C92.0, 15%), alongside CLL (C91.1, 11%), follicular lymphoma (C82.7, 11%) and myeloma (C90.0, 11%). Additional diagnoses show near-universal immunodeficiency (D84.9, 92%) and anaemia in neoplastic disease (D63.0, 82%), with opportunistic mycoses (B48.7, 74%) ranking third. Drug enrichment strongly favours intensive multi-agent chemotherapy with intensive antiemetic and vitamin support drugs (e.g., ascorbic acid +0.11, granisetron, metoclopramide, thiamine, pyridoxine, vinblastine, dacarbazine, doxorubicin, epirubicin, and co-trimoxazole), fully consistent with ABVD and related anthracycline/vinca-based regimens.
- **Community 4—Small, highly treated, high-DDI subgroup.** Community 4 included 31 patients (55% men) who showed the highest drug–drug interaction burden (ADSS 58.84) and the most intensive healthcare utilization (mean 4.84 hospitalizations, LOS 6.41 days) despite only intermediate age (58.48 years) and comorbidity scores (CPS 23.76, Charlson 1.07, Elixhauser 3.41). Principal diagnoses again mix multiple myeloma (C90.0, 16%) with aggressive lymphomas (C82.7 and C83.3, each 13%). Additional diagnoses highlight profound immunodeficiency (D84.9, 90%), anaemia in neoplastic disease (D63.0, 87%), and frequent follow-up encounters (Z11.5, 74%). Over-represented drugs reflect aggressive management of infections and treatment-related complications: furosemide (Δ +0.22), fluconazole (+0.17), ceftriaxone (+0.16), meropenem (+0.12), dexamethasone (+0.15), bisoprolol, ondansetron, metamizole, and yeast probiotics(+0.18)—consistent with repeated cycles complicated by heart failure and volume overload, febrile neutropenia, pain, and nausea.
- **Community 5—Women-enriched, lymphoma-focused chemo phenotype.** The smallest community (n = 18, 67% women, mean age 55.05 years) is markedly women-predominant (67% women) and relatively young (mean age 55.1 years) with intermediate comorbidity (CPS 22.75, Charlson 1.87, Elixhauser 4.30) and moderate DDI burden (ADSS 39.38). Principal diagnoses are dominated by DLBCL (C83.3, 22%) and Hodgkin lymphoma (C81.9, 17%). The most prevalent additional diagnoses are immunodeficiency, unspecified D84.9 (94%), COVID-19, virus not identified U07.2 (89%), special screening examination for other viral diseases Z11.5 (89%). Drug enrichment reflects intensive multi-agent immunochemotherapy and associated supportive care: cyclophosphamide (+0.20), epirubicin (+0.17), vincristine (+0.15), etoposide (+0.14), rituximab, hydrocortisone, etamsylate, potassium chloride, and folic acid—consistent with R-CHOP-like, CHOEP (where etoposide is added to the CHOP regimen), and related regimens delivered to a fitter, lymphoma-focused subgroup.

These data-derived communities show coherent drug and diagnosis enrichment consistent with real-world practice, reinforcing face validity while also generating new hypotheses.

## 4. Discussion

Complex networks have emerged as powerful tools to uncover clinically relevant phenotypes across medical fields—from sleep apnea to cardiovascular cohorts—supporting their use for data-driven clinical grouping in real-world settings [28,29,30]. At the same time, systematic screening for DDIs is crucial for all healthcare providers and highlights the importance of detailed evaluations by pharmacists and the inconsistent agreement found among DDI resources [31,32]. However, the abundance of reported DDI in drug databases contains multiple, inconsistent, or low-evidence listings, which complicates clinical interpretation and network modeling [33]. Our approach mitigates this issue by profiling patients with additional indices (e.g., CPS, Charlson, and Elixhauser) and an aggregate DDI severity score (ADSS); this allows to refine our assessment of DDI burden profiling and better identify associated phenotypes. This way, we emphasize the overall risk signals rather than focusing on isolated pairwise interactions. The present study introduces the Onco-Hem Connectome, a patient similarity network that integrates demographic, comorbidity, polypharmacy, drug–drug interaction burden, and detailed medication exposure data to derive clinically meaningful phenotypes in a real-world cohort of 298 onco-hematological inpatients. Using cosine similarity of rich feature vectors and community detection, we identified five robust patient community-based phenotypes that reflect distinct therapeutic and clinical profiles despite the underlying heterogeneity of hematological malignancies.

The omnibus comparisons support the construct validity of the Onco-Hem Connectome communities. We observed statistically significant and practically meaningful between-community differences for core comorbidity measures: age and mean CPS score (medium $\varepsilon^{2}\approx$ 0.12–0.13), with substantial effects for mean Charlson ($\varepsilon^{2}$ = 0.428) and mean Elixhauser sum ($\varepsilon^{2}$ = 0.650). In contrast, length of stay, number of episodes, and ADSS showed negligible differences after FDR adjustment, with very small effect sizes ($\varepsilon^{2}\approx$ 0.006–0.010). These results indicate that the clustering did not simply recapitulate healthcare utilization or the burden of DDIs but captured broader clinical heterogeneity. The categorical analysis likewise revealed divergent CPS level distributions across OHC communities (Cramér’s *V* = 0.171, small–medium range), reinforcing that the communities clustered along clinically coherent axes of multimorbidity and pain severity. These findings indicate that the PSN clustered patients into phenotypes with significantly different comorbidity burden, consistent with the drug and diagnosis profiles.

### 4.1. Clinical and Pharmacotherapeutic Implications of Onco-Hem Connectome Phenotypes

- **Community 1.** Clinically, this phenotype may inform standardized supportive bundles, including analgesic algorithms, growth factor triggers, and bone health protocols [34,35,36,37,38,39,40,41]. It may also support DDI-aware prescribing, as indicated by ADSS 42, with interaction checks for anthracyclines and targeted agents alongside fluoroquinolones, analgesics, and cardiovascular drugs. Additionally, it emphasizes risk-based monitoring for infectious and cardiovascular complications due to high D84.9 and I10. For responsible management, Community 1 may be a reasonable target for order set optimization (e.g., zoledronic acid + calcium/vitamin D checks; filgrastim criteria; antibiotic de-escalation rules) and drug reconciliation to curb unnecessary adjacent treatments (e.g., routine tramadol) without compromising symptom control [42,43,44]. For prediction purposes, community membership plus core features (CPS, ADSS, I10, D84.9) may help develop phenotype-specific models of prolonged LOS, high-DDI episodes, or infection-related escalation. This approach could facilitate a strategy that prioritizes early prophylactic measures, ongoing interaction monitoring, and targeted supportive care for this mid-complexity subgroup, primarily composed of lymphoma and myeloma patients [45].
- **Community 2.** This phenotype could facilitate a structured approach to prophylaxis and monitoring in clinical settings, including the following components: (i) standardized venous thromboembolism (VTE) and bleeding pathways (implementing dose-adjusted enoxaparin along with renal and platelet monitoring), (ii) infection bundles (using co-trimoxazole and acyclovir based on specific criteria; early escalation to meropenem in high-risk febrile patients), (iii) cardio-oncology co-management (focusing on blood pressure targets, optimizing heart failure (HF) management, and following up on valvular diseases), and (iv) DDI-aware prescribing given the elevated ADSS [46,47,48,49,50,51,52]. For stewardship, phenotype-specific order sets (anticoagulation + antiviral/antibacterial prophylaxis + diuretic algorithms) and interaction watchlists (e.g., QT-prolonging or nephrotoxic combinations) could be built [53,54,55,56]. For prediction, community membership combined with CPS, ADSS, and key ICD codes (I10, I34.0, I50.9, D84.9) may provide a framework for risk models for prolonged LOS, infectious complications, HF decompensation, or 30-day readmission. This approach may enalbe targeted monitoring and earlier intervention for older patients with complex cardiovascular and infectious diseases.
- **Community 3.** This phenotype may support a chemo-toxicity–oriented clinical approach. The most pressing concerns are anticipatory antiemetics, care for mucositis and diarrhea, and proactive electrolyte management due to the frequent occurrence of E87.1 [57,58,59,60,61,62]. DDI-aware prescribing should be prioritized around anthracyclines and antiemetics, with attention to risks related to QT prolongation and metabolic interactions [63,64,65,66,67]. For responsible management, implement triggers for growth-factor administration, criteria for antimicrobial prophylaxis, guidelines for electrolyte administration, and drug reconciliation to avoid redundant treatments. For predictive purposes, community membership combined with core features (CPS, ADSS, E87.1 indicators, and regimen flags) may inform models for predicting febrile neutropenia, infections, unplanned dose reductions, and prolonged LOS [68,69,70,71]. This approach may support the need for early laboratory tests, preemptive supportive care, and timely escalation of treatment.
- **Community 4.** This phenotype highlights patients at particularly high risk of cumulative toxicity and iatrogenic harm, given the intense use of antimicrobials, diuretics, corticosteroids, and cardio-active drugs [72,73,74]. Clinically, the observed pattern may justify structured escalation pathways for suspected infection (early cultures, predefined triggers for broad-spectrum coverage and antifungal stewardship) [75,76,77,78,79,80], alongside cardio-oncology co-management to monitor volume status, heart failure symptoms, and arrhythmia [81,82]. To ensure responsible management, it may be beneficial to standardize order sets that combine antimicrobials, diuretics, antiemetics, analgesics, and electrolyte replacement; include interaction watchlists for QT-prolonging and nephrotoxic combinations; and ensure pharmacist review before each treatment cycle [83,84,85,86,87,88,89]. For prediction, community membership with ADSS, CPS, and key complication markers may help flag risk of high-DDI episodes, antimicrobial escalation, recurrent admissions, and prolonged LOS, which allow for earlier pharmacy intervention and post-discharge follow-up.
- **Community 5.** For this women-enriched phenotype, a pharmacotherapy focused on lymphoma is indicated, including premedication with antiemetics and corticosteroids, neuropathy vigilance for vinca alkaloids, and cardiotoxicity surveillance for anthracyclines [90,91,92,93,94]. Responsible management may include phenotype-specific order sets for CHOP-like combinations, structure DDI screening (anthracyclines, vinca alkaloids, azoles), and monitoring of bleeding risk and potassium balance. For prediction, community membership integrated with CPS, ADSS, and key regimen-adjacent exposures may support models of chemotherapy complications (neutropenic events, electrolyte derangements, cardiotoxicity) and unplanned dose delays; this integration may enable risk-stratified monitoring, timely supportive measures, and coordinated referrals to specialists, where necessary [95,96,97,98,99].

Table 8 translates the five OHC phenotypes into phenotype-specific practice boxes that outline actionable bedside interventions.

### 4.2. Chemotherapy Regimen Signals Across OHC Communities

Regimen patterns delivered a multi-drug perspective that confirmed and contextualized the single-drug over-representation results. Furthermore, these patterns are consistent with the OHC-derived phenotypes. The highest prevalence of VAD is 29% in Community 4, which consists of high DDIs and highly treated patients. ABVD predominated in the chemo-intensive leukemia and lymphoma phenotype (13.1% in Community 3), consistent with its clinical use. The moderate overall prevalence of the R-CHOP regimen, a standard regimen for lymphoma, is probably influenced by patient case mix, the administration of some cycles outside inpatient care, and our stringent detection criteria (e.g., explicit steroid documentation).

Chemotherapy regimen findings strengthen the structure of communities and offer actionable interventions for drug management. These include phenotype-specific order sets, DDI alert lists for anthracycline- and vinca-based regimens, as well as targeted cardio-oncology surveillance when diuretics and antiarrhythmics are co-prescribed.

Our relaxed methodological rule reduces underdetection from real-world medical records while preserving specificity; however, inpatient-only and possibly incomplete pharmacy records may underestimate actual exposure, which may be a limitation of the study. Hence, we emphasize the need for prospective linkage with chemotherapy records from day hospitals and outpatient records in future work.

### 4.3. Study Limitations and Future Work

We conducted a single-center retrospective cohort study, which limits the generalizability due to local practice patterns and documentation habits. We summarized drugs and diagnoses as “ever” exposures at the patient level, so we did not focus on temporal treatment dynamics. These proposed uses (phenotype-specific order sets, DDI watchlists, monitoring priorities) are candidate interventions requiring prospective testing. We did not model the patient clinical outcomes (e.g., mortality or transfers); the clinical utility we present is intended to be hypothesis-generating. Sample size was adequate for network discovery (*Q* = 0.433; within > between similarity, $p<10^{-300}$), but generalization and outcomes linkage require a larger multicenter cohort. While cosine similarity and modularity-based community detection were chosen based on their performance in high-dimensional clinical data [23,25], alternative metrics (e.g., Gower or Jaccard) or clustering algorithms (e.g., spectral or hierarchical clustering) might highlight different facets of patient similarity. Our future studies will consider a multi-metric approach to further validate the stability of these phenotypes. Future activities will also extend to a larger, multicenter cohort, incorporate outpatient chemotherapy records, and carry out prospective trials on phenotype-guided administration and decision support.

## 5. Conclusions

We integrated diagnoses, comorbidities, pharmacotherapy, and DrugBank-derived DDI burden to build the Onco-Hem Connectome (OHC), a patient-similarity network. Community detection on the connected PSN (295/298 patients) identified five phenotypes with robust community structure (modularity *Q* = 0.433). Omnibus comparisons showed significant (FDR-adjusted) between-community differences in comorbidity burden, including age, CPS, Charlson, and Elixhauser indices. Each phenotype showed coherent clinical signatures: (1) a myeloma/lymphoma group that heavily relied on supportive care; (2) an older, multimorbid thrombo-infectious group with anticoagulation and anti-infective enrichment; (3) a younger group with leukemia and lymphoma that required intensive chemotherapy; (4) a small group with high-DDI and extensive treatment; and (5) a women-dominant group exhibiting a pattern of steroid and cytotoxic use. Community-specific drug profiles highlighted over-represented agents and aligned with principal and additional ICD-10 patterns. Chemotherapy regimen prevalence (R-CHOP, ABVD, VAD) further supported the validity of the findings. These phenotypes reflect case-mix and suggest specific stewardship and pathway prospects, such as targeted prophylaxis, DDI monitoring, and supportive care bundles. They also provide a scalable framework for patient-level similarity that can evolve into decision support linked to outcomes over time. Future work will extend to multicenter cohorts, incorporate outpatient chemotherapy records, and test phenotype-guided care pathways in pragmatic evaluations.

## Figures and Tables

**Table 1:** Descriptive statistics of the study cohort (patient-level aggregates).

	Age (Years)	CPS ^1^ (Mean)	Charlson Score (Mean)	Elixhauser Sum (Mean)	ADSS ^2^ (Mean)	Number of Episodes per Patient
Count	298.00	298.00	298.00	298.00	298.00	298.00
Mean	61.91	24.86	1.23	5.77	46.19	3.89
SD	14.66	9.06	1.22	4.65	40.85	2.50
Min	20.00	8.00	0.00	0.00	0.00	1.00
25%	53.00	19.00	0.00	2.35	20.08	2.00
50%	65.00	23.00	1.00	4.00	38.00	4.00
75%	72.96	28.90	2.00	8.00	59.78	6.00
Max	91.33	56.00	5.60	21.33	268.00	12.00

^1^ Comorbidity–Polypharmacy Score. ^2^ Aggregate Drug–drug interaction Severity Score.

**Table 2:** Summary of the characteristics of patients in each community within the Onco-Hem Connectome.

	Community 1	Community 2	Community 3	Community 4	Community 5
Number of patients	99	86	61	31	18
Women (proportion)	0.47	0.44	0.49	0.45	0.67
Men (proportion)	0.53	0.56	0.51	0.55	0.33
Age (years, mean)	63.38	68.26	54.19	58.49	55.05
Length of stay (days, mean)	5.47	6.94	5.16	6.41	6.31
Number of episodes (mean)	3.82	3.64	3.80	4.84	4.33
CPS (mean)	23.88	29.06	21.54	23.76	22.75
Charlson score (mean)	1.10	2.11	0.07	1.07	1.87
Elixhauser sum (mean)	6.79	9.77	0.28	3.41	4.30
ADSS (mean)	41.96	47.87	46.08	58.84	39.38
CPS level 1 (proportion)	0.00	0.01	0.00	0.00	0.00
CPS level 2 (proportion)	0.16	0.02	0.21	0.16	0.17
CPS level 3 (proportion)	0.36	0.23	0.36	0.32	0.39
CPS level 4 (proportion)	0.47	0.73	0.43	0.52	0.44

**Table 3:** Omnibus comparisons across the five Onco-Hem Connectome communities. Continuous variables were tested with Kruskal–Wallis (effect size: $\varepsilon^{2}$) and categorical variables with chi-square (effect size: Cramér’s *V*). False discovery rate (FDR) was controlled with Benjamini–Hochberg within each test family (continuous and categorical).

Variable	Test	Effect Size	FDR-Adjusted *p*	Significant (FDR)
Age (years)	Kruskal–Wallis	$\varepsilon^{2}=0.125$	$6.76\times 10^{-8}$	Yes
CPS (mean)	Kruskal–Wallis	$\varepsilon^{2}=0.123$	$6.76\times 10^{-8}$	Yes
Charlson (mean)	Kruskal–Wallis	$\varepsilon^{2}=0.428$	$1.50\times 10^{-26}$	Yes
Elixhauser sum (mean)	Kruskal–Wallis	$\varepsilon^{2}=0.650$	$2.89\times 10^{-40}$	Yes
Length of stay (mean)	Kruskal–Wallis	$\varepsilon^{2}=0.010$	0.199	No
Number of episodes	Kruskal–Wallis	$\varepsilon^{2}=0.006$	0.322	No
ADSS (mean)	Kruskal–Wallis	$\varepsilon^{2}=0.006$	0.322	No
CPS level	Chi-square	V=0.171	0.023	Yes
Sex	Chi-square	V=0.103	0.532	No

**Table 4:** Top ten drugs per community, their global prevalence, community prevalence, and the largest positive differences (Δ) between community-specific and global prevalence for drugs over-represented in that community (with prevalence ≥ 10%).

Community ID	Drug	Global (Cohort) Prevalence	Community Prevalence	Δ
Community 1	Acetaminophen	0.7	0.74	0.04
	Filgrastim	0.69	0.73	0.04
	Obinutuzumab	0.15	0.17	0.02
	Ciprofloxacin	0.11	0.13	0.02
	Doxorubicin	0.10	0.12	0.02
	Desloratadine	0.67	0.69	0.02
	Tramadol	0.13	0.14	0.01
	Arginine	0.14	0.15	0.01
	Zoledronic acid	0.12	0.13	0.01
	Spironolactone	0.10	0.11	0.01
Community 2	Enoxaparin	0.43	0.56	0.13
	Acyclovir	0.57	0.69	0.12
	Furosemide	0.49	0.60	0.11
	Alprazolam	0.24	0.31	0.07
	Lidocaine	0.27	0.34	0.07
	Sulfamethoxazole+Trimethoprim	0.70	0.77	0.07
	Meropenem	0.10	0.16	0.06
	Metamizole	0.36	0.42	0.06
	Rituximab	0.29	0.34	0.05
	Spironolactone	0.10	0.15	0.05
Community 3	Ascorbic acid	0.78	0.89	0.11
	Metoclopramide	0.19	0.28	0.09
	Vinblastine	0.07	0.16	0.09
	Granisetron	0.75	0.84	0.09
	Dacarbazine	0.06	0.15	0.08
	Doxorubicin	0.10	0.16	0.06
	Pyridoxine	0.77	0.82	0.05
	Thiamine	0.65	0.69	0.04
	Sulfamethoxazole+Trimethoprim	0.71	0.74	0.03
	Epirubicin	0.33	0.36	0.03
Community 4	Furosemide	0.49	0.71	0.22
	Yeast	0.11	0.29	0.18
	Fluconazole	0.64	0.81	0.17
	Ceftriaxone	0.1	0.27	0.16
	Dexamethasone	0.50	0.65	0.15
	Bisoprolol	0.1	0.23	0.12
	Meropenem	0.11	0.23	0.12
	Metamizole	0.37	0.48	0.11
	Ondansetron	0.31	0.42	0.11
	Allopurinol	0.8	0.9	0.10
Community 5	Hydrocortisone	0.59	0.83	0.24
	Cyclophosphamide	0.36	0.56	0.20
	Etamsylate	0.21	0.39	0.18
	Epirubicin	0.33	0.50	0.17
	Potassium chloride	0.17	0.33	0.16
	Coenzyme M	0.06	0.22	0.16
	Rituximab	0.29	0.44	0.15
	Vincristine	0.35	0.50	0.15
	Folic acid	0.07	0.22	0.15
	Etoposide	0.03	0.17	0.14

**Table 5:** The top three principal ICD-10 diagnoses/community in Onco-Hem Connectome (at the patient level). Values in Columns 2–4 are reported as *n*/*N*; %, where *n* is the number of patients with the specified principal diagnosis, *N* is the total number of patients in that community, and % represent the percentage of patients with that principal diagnosis.

Community	First Principal Diagnosis	Second Principal Diagnosis	Third Principal Diagnosis
1	C90.0 (23/99; 23%)	C91.1 (15/99; 15%)	C83.3 (15/99; 15%)
2	C90.0 (19/86; 22%)	C91.1 (11/86; 13%)	C85.7 (11/86; 13%)
3	C92.0 (9/61; 15%)	C91.1 (7/61; 11%)	C82.7 (7/61; 11%)
4	C90.0 (5/31; 16%)	C82.7 (4/31; 13%)	C83.3 (4/31; 13%)
5	C83.3 (4/18; 22%)	C81.9 (3/18; 17%)	C91.1 (2/18; 11%)

ICD-10 code definitions: C90.0—Multiple myeloma not having achieved remission; C92.0—Acute myeloblastic leukemia, not having achieved remission; C83.3—Diffuse large B-cell lymphoma; C91.1—Chronic lymphocytic leukemia of B-cell type not having achieved remission; C82.7—Other types of follicular lymphoma; C81.9—Hodgkin lymphoma, unspecified; C85.7—Other specified types of non-Hodgkin lymphoma.

**Table 6:** Top three most frequent additional ICD-10 diagnoses per community in Onco-Hem Connectome (ever at patient level). Values are reported as n/N (%), where *n* is the number of patients with that additional diagnosis and *N* is the total number of patients in the corresponding community.

Community	First Additional Diagnosis	Second Additional Diagnosis	Third Additional Diagnosis
1	D84.9 (91/99; 92%)	I10 (87/99; 88%)	U07.2 (75/99; 76%)
2	D84.9 (79/86; 92%)	I10 (73/86; 85%)	I34.0 (65/86; 76%)
3	D84.9 (56/61; 92%)	D63.0 (50/61; 82%)	B48.7 (45/61; 74%)
4	D84.9 (28/31; 90%)	D63.0 (27/31; 87%)	Z11.5 (23/31; 74%)
5	D84.9 (17/18; 94%)	U07.2 (16/18; 89%)	Z11.5 (16/18; 89%)

ICD-10 code definitions: D84.9—Immunodeficiency, unspecified; I10—Essential (primary) hypertension; D63.0—Anemia in neoplastic disease; U07.2—COVID-19, virus not identified; I34.0—Mitral (valve) insufficiency; B48.7—Opportunistic mycoses; Z11.5—Special screening examination for other viral diseases.

**Table 7:** Chemotherapy regimen prevalence by community in Onco-Hem Connectome (ever at patient level). Values are reported as n/N; %, where *n* is the number of patients with that regimen and *N* is the total number of patients in the corresponding community.

Community	ABVD	R-CHOP	VAD
1	4/99; 4.0%	9/99; 9.1%	20/99; 20.2%
2	0/86; 0.0%	3/86; 3.5%	15/86; 17.4%
3	8/61; 13.1%	2/61; 3.3%	13/61; 21.3%
4	0/31; 0.0%	2/31; 6.5%	9/31; 29.0%
5	0/18; 0.0%	0/18; 0.0%	1/18; 5.6%

ABVD—doxorubicin, bleomycin, vinblastine, dacarbazine; R-CHOP—rituximab, cyclophosphamide, doxorubicin, vincristine, prednisone/prednisolone; VAD—vincristine, doxorubicin, dexamethasone.

**Table 8:** Practice boxes for each Onco-Hem Connectome (OHC) community: phenotype-specific bedside actions.

Community (Label)	Concrete Bedside Actions
Community 1—Mixed myeloma/lymphoma phenotype with predominant supportive care pattern	Standardize supportive care order set: antiemetics, analgesics, growth-factor triggers (filgrastim), bone-health protocol (zoledronic acid + Ca/vitamin D checks). Monitor infection risk (D84.9), blood pressure (I10), cardiotoxicity of anthracyclines (ECG/QTc, electrolytes). DDI stewardship: anthracyclines, fluoroquinolones, QT-prolonging pairs. Deprescribing: reconcile nonessential adjacent drugs (e.g., routine tramadol) to limit polypharmacy.
Community 2—Older, highly multimorbid thrombo-infectious phenotype	VTE and bleeding pathways: adjust doses of enoxaparin with renal and platelet checkpoints, reversal or escalation rules. Infection bundle: criteria for co-trimoxazole (sulfamethoxazole+trimethoprim) and acyclovir, and early meropenem escalation in high-risk febrile episodes. Cardio-oncology management: blood pressure targets (I10), heart failure optimization (I50.9), and valvular follow-up (I34.0). DDI watchlist: QT-prolonging and nephrotoxic combinations.
Community 3—Younger chemo-intensive leukemia and lymphoma phenotype	Chemotherapy safety bundle: check baseline and interval ECG/QTc, electrolytes ($K^{+}$, ${\mathrm{Mg}}^{2+}$), and neuropathy for vinblastine. Premedication: guideline antiemetics with QT awareness, assess and manage the risk of febrile neutropenia. DDI stewardship: anthracycline, vinca alkaloids, antiemetic agents. Surveillance: early signs of cardio- and neurotoxicity during episodes.
Community 4—Small, highly treated, high-DDI subgroup	High-interaction protocol: pharmacist pre-verification for antimicrobial and cardiometabolic combinations, with renal dosing guidelines. Daily DDI analysis: automated list for QT, nephrotoxicity, and electrolyte-alteration. Monitor electrolytes, renal function, early cardiology consult when escalation (e.g., meropenem, furosemide) occurs. DDI stewardship: culture-guided de-escalation, refinement of diuretic algorithms.
Community 5—Women-enriched, lymphoma-focused chemo phenotype	Steroid management: glucose checks, gastrointestinal protection, sleep hygiene counseling, and taper plans. Hematologic toxicity: complete blood count cadence around cytotoxic and growth factor criteria. DDI stewardship: steroid interactions, avoid additive QT risk. Symptom care: targeted antihistamine and analgesic use to limit polypharmacy.

## Data Availability

The data presented in this study are available upon request from the corresponding author due to the ethical restrictions.

## Abbreviations

The following abbreviations are used in this manuscript:

PSN	Patient Similarity Network
DDIs	Drug–Drug Interctions
CPS	Comorbidity Polypharmacy Score
ADSS	Aggregate DDI Severity Score
ICD-10	International Classification of Diseases, 10th Revision
CAM	Complementary and Alternative Medicines
COPD	Chronic Obstructive Pulmonary Disease
CCI	Charlson Comorbidity Index
IQR	Interquartile Range
OHC	Onco-Hem Connectome
LOS	Length of Stay
CLL	Chronic Lymphocytic Leukemia
DLBCL	Diffuse Large B-cell Lymphoma
VTE	Venous Thromboembolism
HF	Heart Failure
